# Strengthening local capacity for mathematical modelling in low- and middle-income countries: the process and lessons learnt in implementing the first cohort of Nigeria malaria modelling fellowships

**DOI:** 10.1186/s12936-025-05345-2

**Published:** 2025-04-10

**Authors:** Chijioke Kaduru, Uche Ibe, Shina Aladeshawe, Adaeze Eche-George, Ganiyat Eshikhena, Dumale Aadum, Bassey Okon, Emmanuel D. Iorkase, Kesiye Leghemo, Oladipo Ogunbode, Chukwu Okoronkwo, Onyebuchi Okoro, Ehimario U. Igumbor, Abisoye Oyeyemi, Perpetua Uhomoibhi, Seye Babatunde

**Affiliations:** 1 Corona Management Systems, Plot 2014, CAD Zone B09, Celina Ayom Crescent, Kado-Abuja, Nigeria; 2 Bill and Melinda Gates Foundation, Abuja, Nigeria; 3 Bayelsa State Malaria Elimination Program, Yenagoa, Nigeria; 4 Nigeria Centre for Disease Control and Prevention, Abuja, Nigeria; 5 National Malaria Elimination Program, Abuja, Nigeria; 6 Centre for Infectious Disease Research, Nigeria Institute for Medical Research, Yaba, Lagos, Nigeria; 7 School of Health Systems and Public Health , University of Pretoria, Pretoria , South Africa; 8 Niger Delta University, Wilberforce Island, Bayelsa Nigeria; 9 World Health Organization, Abuja, Nigeria

**Keywords:** Malaria modelling, One health, Capacity strengthening, Capacity building, Nigeria

## Abstract

**Background:**

Mathematical modelling plays a crucial role in understanding malaria epidemiology and evaluating anti-malarial interventions. In sub-Saharan Africa, National Malaria Control Programs are increasingly collaborating with modellers to optimize impact within constrained fiscal environments and evaluate the effectiveness of ongoing malaria control efforts. Despite Nigeria’s National Malaria Elimination Program soliciting modelling expertise, there remains a significant capacity gap in low- and middle-income countries (LMICs), including Nigeria. To address this, the Nigerian Malaria Modelling Fellowship (MMF) adopts a one-health approach within the Nigerian Field Epidemiology and Laboratory Training Program.

**Methods:**

The MMF aims to enhance mathematical modelling capacity among Nigerian public health professionals by increasing the number of doctoral and postdoctoral graduates proficient in using modelling for planning, program evaluation, and outcome assessment. This paper highlights the initiative’s innovative aspects and shares initial implementation insights.

**Results:**

Implemented using a human-centred design, MMF is a collaborative effort involving multiple public health stakeholders. The curriculum spans four courses—Malaria, Mathematical Modelling, Evidence Translation, and Project Management—each with targeted modules. The first cohort recruitment attracted 2173 applications, rigorously screened through a five-step process, selecting 33 Fellows from all geopolitical zones of Nigeria. The cohort applies a one-health lens and includes 48% female representation. Key findings highlight the importance of government leadership, gender mainstreaming, stakeholder co-creation, leveraging existing investments, adopting best practices, and expanding engagement to meet national needs.

**Conclusion:**

MMF demonstrates a collaborative effort to build modelling capacity among epidemiologists and healthcare professionals in LMICs, particularly for malaria. The rigorous recruitment process underscores a strong interest in mathematical modelling. The human-centred approach has fostered government leadership, multi-stakeholder engagement, and national ownership. This paper recommends increased commitments to local capacity strengthening in LMICs and advocates for evaluating the project, including assessing Fellows’ competencies post-training to ensure effective capacity development.

## Background

Mathematical modelling (MM) approaches are currently employed to comprehend malaria epidemiology and assess the potential impact of antimalarial interventions [1]. Encouraged by the World Health Organization (WHO) to use MM to support their control efforts [1, 2], the National Malaria Control Programs (NMCPs) in sub-Saharan Africa express a growing interest and are increasingly collaborating with modelling units to shape their National Strategic Plans, enrich their grant applications and evaluate the impact of ongoing control efforts or the potential impact of new interventions [3, 4]. The National Malaria Elimination Program in Nigeria has often sought expertise from the WHO and a few other stakeholders to address modelling-specific challenges. As the country bearing the highest burden of malaria globally [5], and with its commitment to rapidly scale up interventions to achieve national [6] and global targets [7], having sufficient expertise in modelling at the national and subnational levels has become a programmatic imperative.

To bolster the country's capacity in mathematical modelling, the Malaria Modelling Fellowship (MMF) was conceptualized with the primary objective of increasing the number of doctoral and postdoctoral-level graduates with the capacity to employ mathematical modelling to support strategic planning, implementation, monitoring and evaluation of malaria elimination programs at the national and state levels. The initiative, funded by the Bill and Melinda Gates Foundation (BMGF), is a collaborative effort led by Corona Management Systems (CMS)—a nonstate actor—and the National Malaria Elimination Program (NMEP), the Nigerian Centre for Disease Control (NCDC), the WHO, the IQVIA Solutions Pty, which supports evaluation design, and some academic institutions in the country that provide the majority of the teaching staff (moderators and instructors). The fellowship is situated within a government-led health security workforce program—the Nigerian Field Epidemiology and Laboratory Training Program (NFELTP)—which is managed by the NCDC. The NFELTP was launched in October 2008 under the direction of the Federal Ministry of Health (FMoH) and is supported by a multisectoral and multilevel partnership [8]. Accredited by the Training Programs in Epidemiology and Public Health Interventions Network (TEPHINET) [9] the NFELTP trains public health professionals in interventional epidemiology, aiming to increase and coordinate Nigeria’s ability to control infectious diseases and respond to public health threats. The NFELTP has become an important source of skilled health professionals who play a vital role in the control of both infectious and non-infectious diseases in the country [10].

The fellowship delivery employs a human-centred process that integrates ideation, user research, co-creation, rapid prototyping, learning, and iteration. This ensures extensive engagement and consultation in curriculum development and delivery planning, thereby fostering collaboration between skill providers, skill acquirers, and disease control programs. Participants are exposed to a comprehensive curriculum covering core modelling content and in-depth knowledge of malaria epidemiology, including transmission dynamics and adjunct modules that were deemed essential for the fellows’ career. The fellowship aims to produce three levels of disease modelling competencies at the beginner, intermediate, and advanced levels, encompassing different modelling tools and applications.

This Case Study describes the innovative aspects of the fellowship's design and findings from the analysis of resolutions of the MMF working group, reports from project workshops, preliminary activities, and initial formative assessments of fellows based on completed training modules. The success factors and lessons learned from the implementation of planned activities for the first cohort are then presented.

### Structure of the fellowship

The Malaria Modelling Fellowship (MMF) was designed for implementation over a three-year period (2023–2025) and is expected to produce 120 Fellows by the end of the grant. An incremental recruitment approach was adopted, with 30 candidates in the first cohort, 40 in the second, and 50 in the third. Fellows are expected to remain actively engaged in their respective institutions while participating in the fellowship. A mixed delivery method, including a self-paced strategy, is utilized to support candidates in effectively managing both their professional responsibilities and fellowship participation. The fellowship is managed by a Program Management Team (PMT), which oversees operations to ensure its smooth implementation. The roles of the PMT and other key players in the fellowship are outlined in Table 1. The Malaria Modelling Fellowship (MMF) is structured into three phases: inception, fellowship, and close-out.

**Table 1:** List of functions and background of program management team

Program management team	
Functions	Background
• The team formulates and maintains a detailed program schedule, aligning it with project timelines to ensure timely delivery of objectives • Provide technical support and guidance to intending fellows and active fellows • The team monitors and evaluates program performance through established metrics, regularly assessing the success and impact of the program against its predefined objectives • Active feedback collection and analysis from different levels of the malaria modelling fellowship • Effective stakeholder communication is a priority; the team regularly engages with internal and external stakeholders to provide updates, address concerns, and gather valuable feedback • The team exercises keen decision-making skills, making informed choices that align with program objectives and contribute to overall success • The team develops and manages program budgets, providing accurate financial reports and forecasts • Maintaining high-quality standards, implementing rigorous quality assurance and quality improvement processes, and fostering a culture of continuous improvement • Contract and procurement management are handled by the team effectively managing relationships with external vendors and negotiating contracts as necessary • The team ensures legal and regulatory compliance, operating within established frameworks to uphold ethical standards and minimize risks	• Comprehensive expertise in public health, epidemiology, and healthcare within the team • Diverse professional backgrounds contribute to a well-rounded understanding of malaria and infectious diseases • Active engagement with stakeholders at various levels to tailor programs according to unique community needs • Proficient in implementing strategies for infectious disease control, rapid outbreak response, and vaccination campaigns • Recognition of the influential role of policy in shaping public health, leading to active engagement in advocacy efforts • Utilisation of data analytics and surveillance systems to inform evidence-based decision-making • Emphasis on evidence-based approaches to monitor program performance and assess the effectiveness of interventions • Integration of mathematical modelling techniques by a dedicated team member to simulate disease spread and evaluate intervention strategies • Strong quantitative analysis skills contribute to data-driven decision-making, particularly in assessing the impact of public health interventions and program effectiveness

### The inception phase

**Fig. 1:**
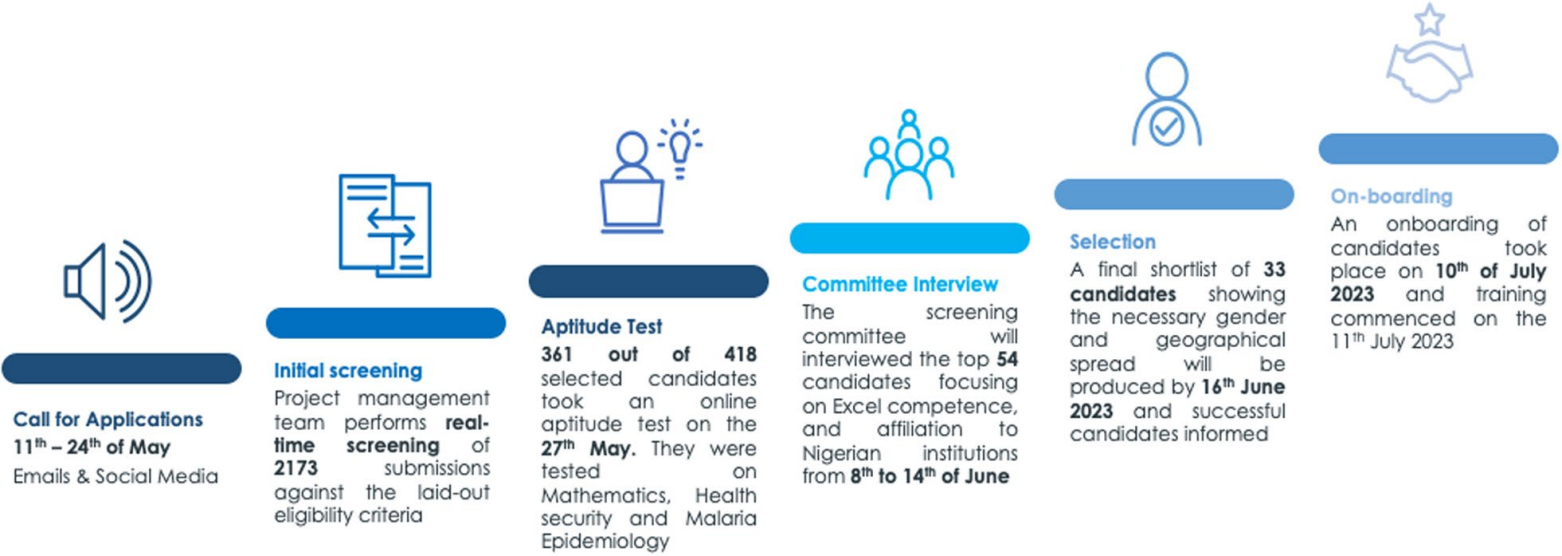
Steps taken in the recruitment of the first cohort of MMF Fellows

This phase involves pre-implementation activities that include preliminary stakeholder engagements with various governments and organized private sector entities involved in the project. At the meetings, the project objectives were extensively discussed, and the stakeholders were aligned on the best approach to address the identified country’s needs for mathematical modelling. Other activities in this phase included curriculum development, alignment with the NFELTP and recruitment of instructors and mentors. Instructors and mentors, drawn from academia and programs, were carefully selected to ensure diverse skill sets catering to prospective fellows who came from different backgrounds. After concluding the essential preliminary activities, the call for applications was made, and this was followed by a selection process that led to the recruitment of the first cohort (Fig. 1). The call for applications was disseminated to public health institutions and programs in one health ecosystem, and the staff who were eligible for the fellowship were encouraged to apply. The call for applications was also shared with the public through social media platforms (LinkedIn, Twitter, and Instagram), and the call was also disseminated to Nigerian Institutions through institutional email addresses.

The following eligibility criteria centring on education, experience, and skills were developed and finalized during an inception workshop where a consensus was reached by MMF working group members on a number of important elements of the fellowship.

#### Education

The essential requirement was possession of a master’s or higher degree in public health, epidemiology, biostatistics, bioinformatics, or applied mathematics or having specialized training in malaria or other infectious disease control programs. The latter applied to residents and graduates from accredited field epidemiology training programs. The desirable qualification was specialized training in quantitative data analysis.

#### Experience

Applicants were essentially required to have a current affiliation with a Nigerian public institution working in one-health (human health, animal health, and the environment) and a minimum of 3 years of professional experience. Applicants with the following qualifications were also considered: (i) postdoctoral or junior lecturer in epidemiology or biostatistics; (ii) residency in public health/community medicine; (iii) data management expertise from the malaria program, National Stop Transmission of Polio, rapid response team, or other emergency preparedness and response team; and (iv) expertise in quantitative data analysis in monitoring and evaluation with involvement in Ministries, Departments and Agencies (MDAs) working across the one-health ecosystem.

#### Skills

Requirements included proficiency in the use of Microsoft Excel, effective communication skills in verbal and written English and demonstrable problem-solving skills with the ability to work effectively with minimal supervision.

**Fig. 2:**
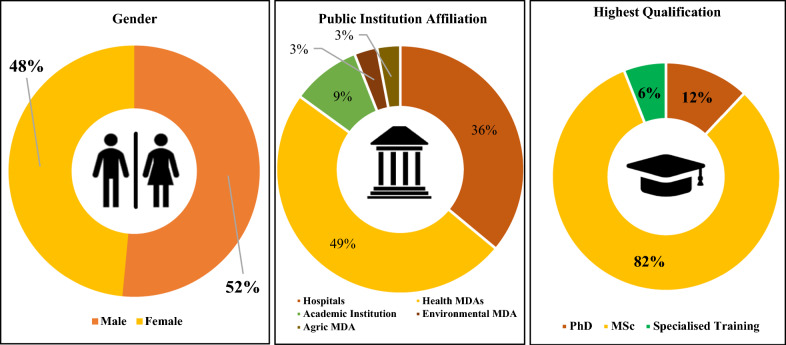
Overview of the profile of cohort 1 fellows (1). *MDA *Ministries, Departments and Agencies, *CMS* Corona Management Systems

Other criteria considered in the selection process included gender balance, need for geographical representation of the country’s six geopolitical zones, strong institutional affiliations using a one-health approach and age of candidate, prioritizing individuals younger than 50 years to ensure sustained knowledge transfer to national institutions and supporting younger early-career researchers. Thirty-three candidates were eventually included in the first cohort. The application of a gender lens in the selection process resulted in an almost balanced sex representation, with a slight male preponderance (17/33, 52%). Most of the fellows (14/33, 42%) were employed in public health/community medicine residency training programs in tertiary hospitals, and the majority (27/33, 82%) had a master’s degree (Fig. 2). All the participants had a science background, with 20% of them having a degree in pure mathematics or biostatistics.

**Fig. 3:**
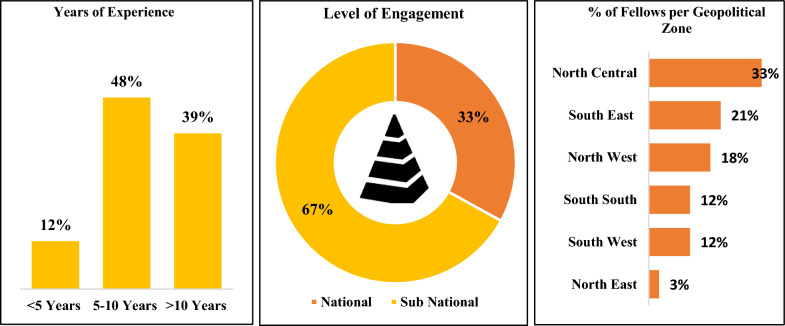
Overview of cohort 1 of fellows (II)

Figure 3 shows that close to 90% of the cohort 1 fellows had a minimum of 5 years of work experience at admission, and two-thirds of them (67%) had affiliations with subnational-level malaria and other disease control institutions. The North Central Geopolitical Zone (NCZ), where the Federal Capital Territory (FCT) is located, had the highest proportion (33%) of intake, reflecting a concentration of fellows affiliated with national-level agencies in the FCT. This high proportion was due to the number of applications received from the zone. Most of the national programs and institutions where mathematical modelling skills are needed are based in FCT. The low proportion (3%) of representation from the Northeast reflects the disproportionately low number of applications and qualifications of applicants from the zone based on the eligibility criteria.

### The fellowship phase

This phase consists of three stages and spans a 10 month period per cohort (Table 2). The three stages—beginner, intermediate, and advanced—comprise 310, 195, and 135 h of learning, respectively. It involves in-person and remote didactic and applied sessions, tutorials, assignments and self-directed learning. The curriculum development process incorporated expert opinions from stakeholders, iterating on modules, delivery approaches and evaluation questions. The finalized curriculum outlining the fellowship structure was made available online for prospective candidates at the time the fellowship was advertised. Stakeholder consultations and user feedback occur at each stage, guiding curriculum iterations. Tables 3, 4, 5 detail the notional hours for the fellowship (the expected learning time required by the average student to accomplish the course unit) and the fellowship component courses and their duration, while Table 6 provides an extended summary of the description of each course. Initial formative assessments through pre- and post-tests indicate increased knowledge across malaria and modelling courses.

**Table 2:** Overview of notional hours for the Fellowship

Learning modality	Notional learning hours		
	Beginner	Intermediate	Advanced
Lectures (In-person/face-to-face)	80 h	135 h	240 h
Online learning and self-paced learning	100 h	190 h	300 h
Tutorials/group discussions	30 h	60 h	60 h
Independent study hours	50 h	60 h	70 h
Assignments	50 h	60 h	70 h
TOTAL	310 h	505 h	640 h

**Table 3:** Overview of the modules in the Malaria course component of the fellowship

Code	Duration (120 h)	Course
MFMAL 101	15 h	Introduction to malaria
MFMAL 102	30 h	Malaria vector biology and control
MFMAL 111	15 h	Case management of malaria
MFMAL 121	15 h	Malaria chemoprevention
MFMAL 131	15 h	Malaria bioinformatics
MFMAL 141	15 h	Malaria social and behaviour change communication
MFMAL 151	15 h	Malaria program & organisation management

**Table 4:** Overview of the modules in the Modelling course component of the fellowship

Code	Duration (480 h)	Course
MFMOD 103	45 h	Microsoft excel for disease modelling
MFMOD 102	30 h	Mathematical background I
MFMOD 105	75 h	R for disease modelling
MFMOD 205	75 h	Introduction to python for scientific computing
MFMOD 202	30 h	Overview of infectious disease modelling
MFMOD 203	45 h	Stochastic disease modelling
MFMOD 212	30 h	Mathematical background II
MFMOD 213	45 h	Structured population dynamic models
MFMOD 223	45 h	Compartmental model of disease dynamics
MFMOD 301	15 h	Mathematical background III
MFMOD 303	45 h	Agent-based modelling

**Table 5:** Overview of the modules in the Project management and evidence translation course components of the fellowship

Code	Duration (40 h)	Course
MFPOM 101	10 h	Project management in a box
MFPOM 111	4 h	Overview of International Health and Global Health Financing landscapes
MFPOM 121	2 h	Gender mainstreaming in Public Health
MFPOM 131	2 h	Physical and Sexual Exploitation and Abuse
MFETL 101	4 h	Research methods and literature reviews
MFETL 111	4 h	Grantsmanship
MFETL 121	4 h	Scientific writing and manuscript development
MFETL 131	4 h	Leadership of evidence translation
MFADJ 101	Additional elective courses (up to 2 courses)	• An Introduction to Thinking and Working Politically; or • Introduction to Ethics in Evidence Generation (Basic) • Social Norms Programming and Measurement; or • Health Economics and Financing (Basic)

**Table 6:** Detailed summary of course descriptions

S/N	Courses	Description	Module aims
1	Introduction to Malaria	This course introduces malaria, Topics covered include the life cycle of the malaria parasite, its transmission, and current control strategies. The course explored the history of malaria, its prevalence around the world, and the global efforts to eradicate it. Additionally, the course will discuss the impact of malaria on human health and the environment, as well as the potential for new treatments, intervention, and prevention strategies	• Causes and Epidemiology of Malaria • Local and Global Burden of Malaria • Basic Principles of Malaria Control and Prevention • The Control to Elimination Spectrum for Malaria Interventions
2	Malaria vector biology and control	The "Malaria Vector Biology and Control" course provided an in-depth understanding of the biology, behaviour, and control strategies of mosquito vectors responsible for transmitting malaria. This course focused on the key vector species, their life cycles, host-seeking behaviours, and the ecological factors influencing their population dynamics. Additionally, students explored various vector control interventions and their effectiveness in reducing malaria transmission	• Introduction to Malaria and Vector-borne Diseases • Malaria Vector Species • Mosquito Life Cycle and Behavior • Vector Control Strategies, Overview and Implementation
3	Case management of malaria	This course was designed to give an overview of the complexities of managing malaria cases. It covered the basics of diagnosing and treating malaria and discuss the evolution of the current treatment guidelines and trends in malaria control and case management strategies	• Malaria case definitions & terminologies • Recommended approaches for malaria diagnosis • Malaria treatment modalities and service delivery • Parasite sentinel surveillance and therapeutic efficacy studies
4	Malaria chemoprevention	The "Malaria Chemoprevention" course provided students with a comprehensive understanding of the strategies and approaches used for chemoprevention of malaria. Chemoprevention involves the use of antimalarial drugs to prevent or reduce the occurrence of malaria infection in populations at risk, particularly in endemic regions. This course explored the different chemoprevention approaches, including intermittent preventive treatment (IPT) and seasonal malaria chemoprevention (SMC), their implementation, effectiveness, and challenges. Through lectures, case studies, and interactive discussions, students explored the evidence base for chemo-preventive interventions, learnt about monitoring and evaluation techniques, and develop an appreciation for the complex factors influencing the success of chemoprevention programs. By the end of the course	• Importance of chemoprevention as a strategy for malaria control • Mechanisms of action and the development of drug resistance • Safety and efficacy considerations in the use of antimalarial drugs • Explore strategies for implementing IPT and address associated challenges • Concept of SMC and its application as a targeted chemoprevention strategy • Surveillance systems for adverse events and drug resistance monitoring
5	Malaria bioinformatics	This course provides students with an understanding of the basic principles of bioinformatics and how they are applied to the study of malaria. It covered topics such as data collection and analysis, and the use of bioinformatics tools and techniques for malaria research. The fellows learnt about malaria-related databases, tools, and software, and be able to identify bioinformatics resources and assess their usefulness	• Overview of surveillance, Monitoring and Evaluation (SME) in health • Contextualizing SME for Malaria Program • Introduction to the National Health Management Information System (NHMIS) & District Health Information system
6	Malaria social and behaviour change communication	This course introduced Malaria Social and Behaviour Change Communication (SBCC) and provided fellows with an understanding of the social and behavioural context of the disease and its impact on the populations most affected. Students learnt how to use communication strategies to influence social and behavioural change in relation to malaria prevention, control, and treatment. Fellows gained knowledge of the different approaches to SBCC, including social marketing and health promotion campaigns in Nigeria. The course explored the ethical considerations of SBCC, the use of technology and digital media, and the importance of monitoring and evaluation. The course hinged on case studies and examples to in Nigeria to illustrate the knowledge and skills acquired	• Introduction to SBCC: Principles and Theories • The National SBCC strategic Framework for Malaria • Monitoring and Evaluation of Malaria SBCC • Malaria stakeholder Landscape and Engagement in Nigeria
7	Malaria program & organization management	The purpose of this course is to provide fellows with the knowledge and skills to effectively manage and implement a malaria program and organization. Fellows will learn how to design and implement a comprehensive malaria control and elimination strategy, develop an effective malaria monitoring and evaluation system, and understand the impact of malaria on different populations and communities. In addition, the course will cover the importance of effective communication and collaboration between different stakeholders, and how to effectively manage resources and budgets	• Health Management and Policy Development in Nigeria • National Malaria Response in Nigeria • Overview of Global & National Policy & Guidelines for Malaria • Developing of Malaria Strategic Planning & Review processes for Malaria Programs • Acquire competence in the use of Organizational Capacity Assessment & Development tools for malaria
8	Mathematical background I	This course was designed to provide students with the necessary mathematical foundation and skills required to excel in various fields of science and modelling. The course covered a wide range of mathematical topics, from basic concepts to more advanced techniques, enabling students to develop a solid understanding and application of mathematical principles in their respective disciplines. Through a combination of theoretical lectures, problem-solving exercises, and practical examples, students will the confidence and proficiency needed to tackle complex mathematical problems and apply mathematical tools effectively	• Fundamentals of Mathematics • Algebra and Functions • Trigonometry • Analytical Geometry • Calculus Differentiation, Integration • Differential Equations • Probability and statistics • Linear Algebra
9	Introduction to excel for modelling	This course is designed as the first encounter to scientific computing for the fellows. It would set the tone for the rest of the modelling course but allowing the student to use excels cell position relations to solve equations and predict parameters without writing codes	• Basic Matrix Operations with Excel (MMULT, MINVERSE, TRANSPOSE etc.) • Numerical optimization with excel • Euler method of solving differential equation using excel • Curve fitting with excel • Solution of Compartmental SIR model with excel • Data Matching and Parameter estimation with excel

### Transition and close-out phase

This phase involves the conclusion and presentation of an individual mathematical modelling project (capstone project) by the fellows, an end-term evaluation, key learning product development, and the production of an end-term report. The capstone projects aligned with the NMEP and NCDC research priorities and covered malaria and other infectious diseases. They will be included as key learning products to be shared during the close-out phase. The projects were supervised by mentors (one mentor to a fellow), and a committee reviewed each fellow’s final project (Table 7). The committee consisted of an external supervisor with a modelling background, an internal modeller from the program team, the co-principal investigator of the project, an independent consultant who provided technical support for the program, and representatives from the NMEP and the NCDC. The Fellows are expected to demonstrate competency at the end of each of the 3 phases of the fellowship. This is done through the capstone projects at each phase, where Fellows conduct research and build policy-relevant models addressing challenges in control efforts towards malaria and other infectious diseases. After review by mentors, the Defence Committee, and the collaborating institutions, the Fellows are expected to produce manuscripts for publications in peer-reviewed reputable journals.

**Table 7:** Average knowledge increase among fellows

Course	% Knowledge increase (%)
Introduction to Malaria	25
Malaria vector biology and control	66
Case management of Malaria	39
Malaria chemoprevention	53
Malaria bioinformatics	46
Malaria social and behaviour change communication	38
Malaria program & organization management	81
Mathematical background I	73
Introduction to excel for modelling	50
Program management in a box	89
Foundations of research methodology	86
Linear algebra theory	85
Differential equation	75
Probability of statistics	89
Emerging health challenges	89

Table 8 lists the approved capstone project topics for the beginner stage. A detailed plan will be developed to ensure that all data collection methods and evidence generation principles for the program are clearly explained and transferred to government staff, who serve as custodians of the NFELTP program, to support the continuity of interventions. The transition of interventions through capacity building and the gradual transfer of responsibilities will be fully initiated twelve months before the project's closure to facilitate a smooth transition and provide sufficient hand-holding support by the implementation team.

**Table 8:** List of finalised fellow-led malaria modelling Capstone projects

S/N	Thematic area	Capstone projects
1	Malaria	Derivation relatively technique parameter inference malaria disease
2		Modelling the impact of IRS on malaria prevalence in Nigeria
3		Modelling the effect of health interventions on malaria prevention
4		Modelling the effectiveness and non-use of long-lasting insecticidal nets LLINs
5		Assessing the impact of vector control strategies on malaria incidence
6		Modelling the effect of insecticide-treated nets on malaria transmission in Sakaba LGA, Kebbi State, Nigeria
7		Spatial Modelling of vector breeding sites and their effect on malaria transmission
8		Modelling the effects of seasonality on malaria incidence
9		Modelling the effect of seasonality on the incidence of malaria: a case study of Osun State
10		Modelling the effects of conflict and displacement on malaria burden
11		Effect of the new guidelines on chemoprevention in pregnancy on the incidence of malaria amongst pregnant women in Enugu state, Nigeria
12		Static spatial modelling of malaria cases in Nigeria
13		Modelling Age- structured malaria transmission dynamics in Kebbi State
14		Modelling the effect of yearly SMCs in supported areas and the impact of large- scale deployment
15		Modelling the impact of seasonal malaria chemotherapy (SMC) drug dosage compliance on malaria spread among infants
16		Modelling the effectiveness of perennial malarial chemoprevention on malaria prevalence
17		Modelling the basic reproduction number (R0) of malaria in different regions
18		Modelling the benefits of testing before treatment in Malaria over syndromic management
19		Estimating the effects of malaria control interventions on malaria transmission in Nigeria: a mathematical modelling approach
20		Assessing the role of human behavior in malaria outbreaks
21		Modelling the effects of current malaria control interventions (ACT, LLIN) on malaria prevalence to inform malaria control interventions in FCT Abuja, Nigeria
22	Infectious diseases	Modelling the Diphtheria outbreak in FCT
23		Socio-cultural and Environmental Risk Factors associated with outbreak of Lassa fever in high burden communities in Nigeria
24		Modelling the prevalence of Lassa Fever in Nigeria
25		Socio-cultural factors influencing the prevalence of Cholera in the Federal Capital Territory (FCT), Nigeria
26		Cholera hotspot mapping in Nigeria
27		Prevalence of Measles among children under 9 months in Nigeria
28		Evaluation of Monkey pox surveillance system in Nigeria
29		Modelling the prevalence of Yellow Fever in Nigeria
30		Modelling the trend of Diphtheria morbidity and mortality in Nigeria

### Evaluation and impact assessment of the fellowship

In evaluating the program, the importance of gathering user feedback was recognized to gain comprehensive insights into the impact and effectiveness of its initiatives. The approach focused on direct engagement with fellows and all stakeholders to understand their experiences and perspectives on various aspects of the program. A structured feedback mechanism was designed, incorporating structured surveys to capture diverse viewpoints. These activities were carefully tailored to elicit specific feedback on elements such as content relevance, delivery methods, and overall impact on professional development. As a result, valuable insights were obtained, informing enhancements and refinements to the program. The feedback loop facilitated the identification of challenges, refinement of program content and delivery schedules based on user preferences, and implementation of targeted interventions to maximize the program's impact.

The main evaluation activities were:

- Pre and post-tests administered before and after course delivery to assess knowledge change (increase, static and gaps). Specific topics with the most knowledge gaps were identified and targeted support was provided to Fellows to enable them to grasp the concepts.
- Course evaluation that contained close- and open-ended questions through which the fellows assessed the delivery of the course. This assessment reviewed the course relevance, their perceived experience of the instructors, and their perception of the assessments. The open-ended questions yielded feedback that provided a more holistic understanding of their learning experience.
- The capstone projects, which also contributed somewhat to the impact assessment as the Fellows demonstrated their modelling competency through research and defence of the projects before a Defence Committee made up of subject matter experts in infectious disease control and mathematical modelling.

The evaluation plan includes following up fellows after completion of the course and returning to their organization to assess their behaviour, in this case the application of their modelling expertise to their work, and the results to the organization’s mission, thus completing Kirkpatrick’s training evaluation model**.**

## Lessons learnt

This is the first modelling fellowship of its kind that was organizedd in Nigeria. The key lessons and critical success factors identified across the inception and initial fellowship phases are summarized below.

### Success factors and lessons learnt from the first cohort of malaria modelling fellowships

#### Government leadership

The approach to the delivery of this fellowship has been to have the sustained buy-in and leadership of the program by the NMEP. NMEP has led every public event on the fellowship, provided leadership of the cocreation process and is ensuring that the fellows understand the research needs of the disease control program. There has also been very strong leadership from the NCDC and the NFELTP unit of the NCDC. This leadership is key to the co-creation approach and ensures that modelling outputs from the fellows will respond to disease control intervention needs of the country. Currently, the Malaria Modelling Fellowship is largely perceived as an NMEP and NCDC program supported by partners.

#### CO-creation

The human-centred design (HCD) approach ensures that the voices of stakeholders from the government, nongovernmental organizations, the WHO and academia, as well as those of prospective and now recruited fellows, have been instrumental in the wins recorded thus far. The HCD has created a sense of ownership among a diverse group of stakeholders and has increased accountability in the delivery of the fellowship. The stakeholders participate in regular quarterly cadence that has been instituted to maintain alignment and sustain buy-in. They have also been critical in identifying resource persons from academia and in shaping the operational research lines of enquiry around which the capstone projects were designed by the fellows.

#### Building on existing investments

The fellowship is domiciled in the NFELTP unit, building on all the investments that had led to Nigeria establishing a field epidemiology training program. Additionally, working very closely with the NCDC, the existing NCDC-owned online learning management system (LMS) was redesigned for scale to support the overall delivery of MMF courses and to serve as an archive of all available learning resources. The LMS was initially built with support from the Bill and Melinda Gates Foundation and other partners, and it was important to build incrementally on that previous investment. The LMS was optimized to provide learning materials, administer assessments, and enable submission of assignments from the Fellows. The redesign effort was such that the LMS would support multiple capacity building programs such as the MMF. The case study here is a successful example of building on existing investments.

#### Gender considerations

From the initial advertorials for the fellowship, clear communication was established to encourage female candidates to apply. This communication emphasized a commitment to supporting eligible women with children under 18 months by providing assistance with transportation, accommodation for their children and childminders, or access to daycare services as needed during in-person training and field postings. This gender-sensitive approach is believed to have contributed to the 48% female representation in the first cohort of Fellows.

#### Leveraging best practices

The fellowship has been designed to leverage best practices in the design and implementation of workforce capacity development, including stepwise screening and selection processes and the use of a mix of adult learning techniques. The selection process included an initial screening using the eligibility criteria, an online aptitude test, an interview with the screening committee, a Microsoft Excel-based assessment, and a final shortlisting and validation of the final shortlist. Candidate selection was managed by a screening committee consisting of professionals from the malaria program, academia, and the health security system to ensure a comprehensive approach to the selection of Fellows for a successful program.

#### Accreditation

The fellowship is designed as a nondegree-awarding competency-based training. The depth of the curriculum and the qualifications of the course instructors and mentors drawn from academia and disease control programs have, however, necessitated discussions on the potential benefits of an academic institution-led certification and accreditation. This important lesson can inform future competency-based training for public health practitioners.

#### Widened stakeholder pool

Based on the lessons from this round, there are plans to specifically engage with the National Primary Health Care Development Agency (NPHCDA) for future cohorts. Health and disease modelling also played a key role in supporting National Immunization Technical Advisory Groups (NITAGs), vaccine introduction, vaccination campaigns, and the wider immunization program. Deepening competency in a targeted manner in that ecosystem will be a logical, deliberate step when recruiting the next cohort.

These success factors and lessons learnt highlight the importance of government leadership, collaboration, adaptability, and inclusivity in designing and implementing effective public health capacity-building programs.

### Challenges

Executing the fellowship presented a set of challenges that could be regarded as limitations initially but offered opportunities for reflection and refinement of the implementation methods. Addressing these challenges was essential to ensuring the program’s success. The encountered challenges and their corresponding mitigation strategies are outlined below:

#### Disruptions to the course timetable and schedule

The fellowship experienced a few disruptions to the planned modular approach of delivering the course contents. These were due mainly to uncontrollable issues such as flight delays and eleventh-hour changes in the schedules of the course instructors or moderators. The program team envisaged these and was able to develop a contingency plan in collaboration with the course instructors and moderators. For instance, instructors who were not based in the city where physical lectures were delivered were made to send a recording of their lectures ahead, and this was used where necessary. Others had to give their lectures virtually when on-site delivery was not feasible. This allowed for flexibility in the delivery of the courses while still ensuring the modular approach.

#### Data management for capstone projects

In line with the program objective of tailoring the outcomes of the modelling capstone projects to target the country’s institutional needs and provide research support, the program encountered nonavailability of quality data across the different thematic areas to support the development of models for those research pieces. The program aligned with the stakeholders and had a consensus to restructure the research questions to enable fellows to work with the available data while preserving the original concept of the research. To address data availability challenges, the fellowship actively involved government agencies. The inclusion of the NMEP in the program ensures that government institutions provide essential administrative support, access to survey data, and other necessary documents for modelling studies. This collaborative approach facilitates improved data access and enhances the overall quality of research.

#### Conflicting schedules for fellows and instructors

The program experienced challenges with scheduling training sessions due to the conflicting free times of both the fellows and the instructors. Fellows and instructors were seen to express difficulty attending regular sessions due to their job roles, which demand flexibility in hours. Instructors, on the other hand, mention their packed schedules, including consultancy work and research commitments. The program team mitigated these issues by sharing flexible proposed program schedules ahead of time to give fellows and instructors ample time to plan attending training sessions. Furthermore, the team also ensured personal follow-ups with each instructor and fellow to reconfirm their availabilityatf proposed timelines for training sessions.

### Delay and unavailability of requested data for capstone projects

An integral component of the program involves the completion of a capstone project at each stage. Fellows are required to regularly access data from databases maintained by government institutions. However, delays were encountered in obtaining these data from the relevant agencies. To address this issue, the program management team devised ensured that the topics for the capstone projects were aligned with the research agenda of the government agencies that could benefit from modelling support. Subsequently, a comprehensive request was sent to these agencies to gather the necessary data for developing research models.

## Conclusion

The Malaria Modelling Fellowship (MMF) is a collaborative effort to build mathematical modelling capacity among epidemiologists and other infectious disease control professionals in low- and middle-income countries (LMICs). This initiative aims to increase the number of modellers based in African institutions. The recruitment process underlines a strong interest in mathematical modelling, and the human-centred design approach facilitates government leadership, multi-stakeholder involvement, and country ownership. Delivering the first cohort has been challenging, but lessons have been learnt that would make the subsequent cohorts easier to manage. There will be a holistic evaluation of the project, including an assessment of the capacity of Fellows upon completion of the training to determine how the capacity development translates to the expected competency and application in their workplaces. There is a need for continuous stakeholder involvement and sustained support in building and strengthening local capacity in mathematical modelling in LMICs where infectious diseases are still a major threat to public health.

The program has secured stakeholder buy-in and ownership from the National Malaria Elimination Program (NMEP), the apex malaria control institution in Nigeria. The malaria research projects are vetted by the NMEP to ensure that there is alignment with its National Malaria Operational Research Agenda (NMORA), which contains priority research areas in malaria. Additionally, the NMEP is involved in the defence activities for the capstone projects to provide oversight on the directions for research and development. Also, the modelling program is designed to ensure strong representation of individuals from government institutions within the cohorts. This approach empowers them to drive research priorities, enhance the utilization of evidence, and support data-driven decision-making based on modelling insight.

## Data Availability

Data is provided within the manuscript and the supplementary information files.
